# Special screw corridors and imaging in pelvic ring trauma

**DOI:** 10.1007/s00402-024-05610-0

**Published:** 2025-01-04

**Authors:** Axel Gänsslen, Jan Lindahl, Richard A. Lindtner, Dietmar Krappinger, Mario Staresinic

**Affiliations:** 1 Department of Trauma Surgery, Hannover Medical School, Hanover, Germany; 2 Johannes Wesling Klinikum Minden, Minden, Germany; 3 Helsinki University Hospital, Helsinki, Finland; 4 Innsbruck Medical University, Innsbruck, Austria; 5 University Hospital Merkur, Zagreb, Croatia

**Keywords:** Pelvic ring injury, Bone corridors, Imaging, Radiographic anatomy

## Abstract

Adequate intraoperative visualization is mandatory for implant application in pelvic ring injuries. Several fluoroscopic X-ray views are in practical use. The gold standard primary X-ray is the anteroposterior view of the pelvis. In addition to this view, oblique views for pelvic ring instabilities and acetabular fractures are well defined. Combinations of these views allow better identification of osseous corridors for screw applications. These corridors are based on the 3-ring concept of the hemipelvis. For pelvic ring stabilization the main osseous corridors include the retrograde and antegrade superior ramus/anterior column corridor, the supraacetabular corridor and the gluteus medius pillar corridor. The radiographic anatomy of these corridors is described in detail for screw applications with definition of image intensifier angulations, risk zones and corridor parameters. This allows for intraoperative safe implant application.

## Introduction

The intraoperative gold standard to analyze the results of pelvic ring reduction and fixation is based on several fluoroscopic X-ray views.

The gold standard primary X-ray is the anteroposterior view of the pelvis (PAP). In addition to this view, no true radiological perpendicular plane is available as a true lateral view (TLV) is hard to analyze in fracture situations due to overlapping of both hemipelves.

Analysis of the pelvic ring can be performed by additional oblique views allowing better understanding of the ring structure of the pelvis and a better displacement analysis of pelvic ring injury deformity. Thus, additional “pelvic views” include the Pelvic Inlet (PIV) and Pelvic Outlet (POV) views which were introduced by Pennal et al. [48].

In the true frontal plane of a PAP, the obturator segment and the iliac wing have an perpendicular orientation. Thus, oblique views are taken for two-plane analysis with the pelvis rotated either right or left by 45°. These views were optimized and described by Judet based on ideas of Waller [35, 37, 38, 73].

These “acetabular” views allow optimal comparison to the opposite side when the whole pelvis is visualized. The oblique views consist of the Obturator Oblique View (OOV) and the Iliac Oblique View (IOV).

Combination of “pelvic ring” and “acetabular” views often represent the basis for bone corridor analysis in pelvic ring and acetabular fracture situations.

These views (PAP; OOV and IOV (so-called Judet views), PIV and POV (Pennal views)) are the basis of evaluation of pelvic ring and acetabular injuries as different bone corridors allow analysis of fracture location, displacement, intraosseous hardware, and intra- and postoperative results.

Pelvic and acetabular oblique views can be combined for relevant intraoperative visualization of special bone corridors.

**Table:** 

Definition	
When a combination of Pennal views and Judet views is performed, the first character is a “C” representing the term “combined”. It is followed by the Judet-part of the view (always two characters e.g. OO) and the Pennal type (one character, e.g. O (Outlet)) of the view: COOO = Combined Obturator Oblique Outlet view)	

Understanding intraoperative imaging for implant placement in special screw and implant corridors depends on understanding of the radiological projections and the basic bony anatomy of the hemipelvis.

## Hemipelvic ring theory

**Fig. 1:**
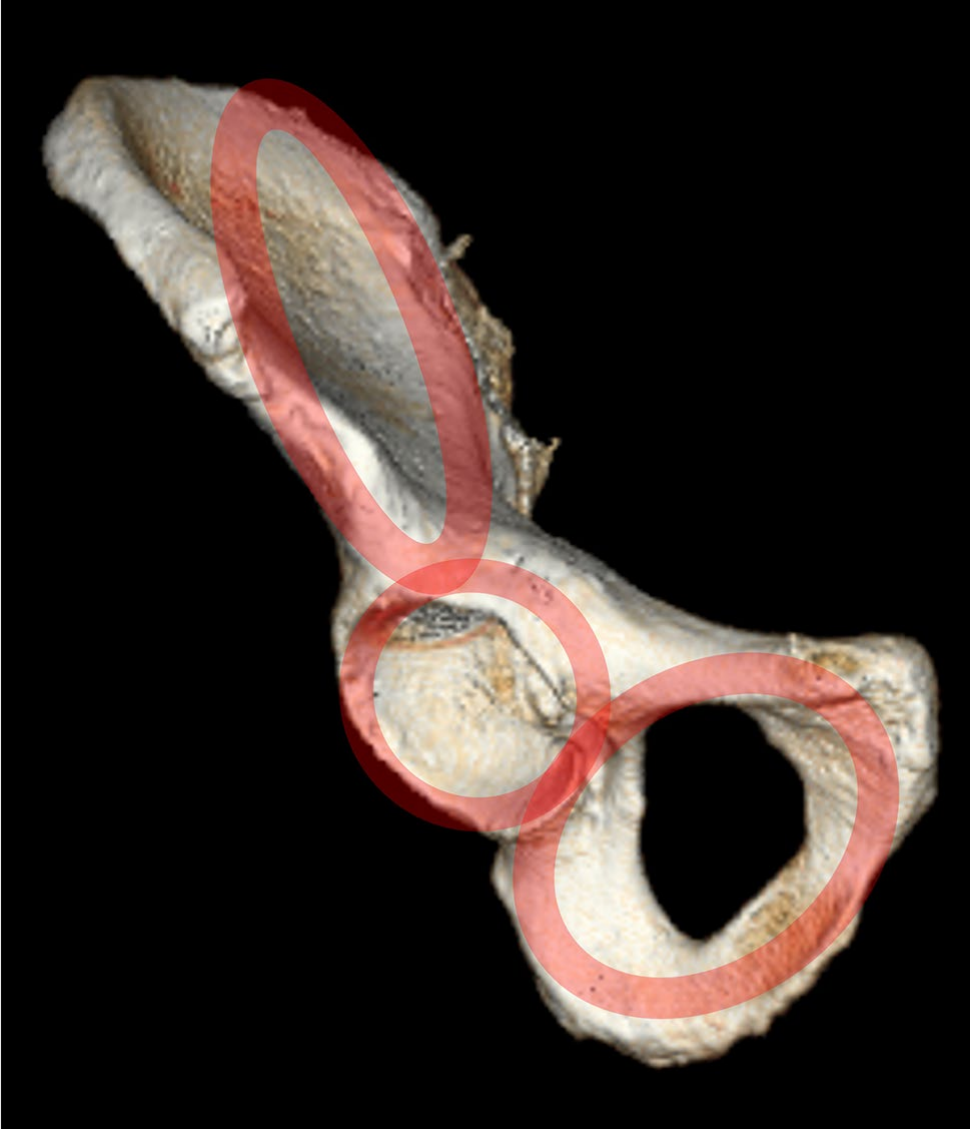
The hemipelvis consists of 3 rings, relevant for adequate implant positioning: iliac ring, acetabular ring, obturator ring

The hemipelvis can be interpreted as a three-ring structure [22] consisting of (Fig. 1):

- Iliac ring
- Acetabular ring
- Obturator ring

**Fig. 2:**
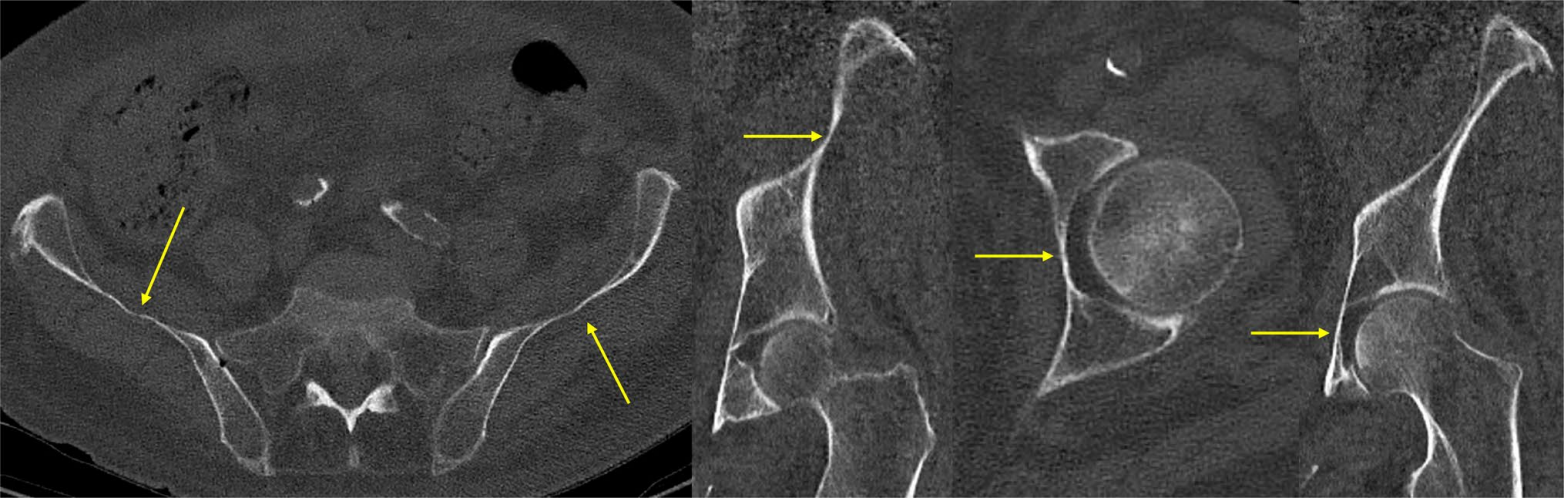
Anatomy of the central ilium (left two pictures) with an extreme central thinning. Central, medial part of the acetabulum (right two pictures) with thinning of the quadrilateral plate

In each ring, centrally there is no or only a reduced amount of bone. While the obturator ring is already a true ring, both the acetabular and iliac fossa ring structure have still some small central bone, which is maximally 2-3 mm in thickness (Fig. 2). In Chinese people, already the acetabular ring shows sometimes a real ring structure [21].

Thus, the periphery of these ring structures is suitable for adequate implant positioning in pelvic ring and acetabular fracture surgery (Fig. 1).

**Table:** 

The hemipelvis consists of three overlapping rings	

### Iliac ring

The special feature of the iliac wing segment consists of a ring-shaped structure with a very thin central bone plate central surrounded by dense bone. Anterior, within this ring structure there is a bony condensation, the gluteus medius pillar. Between the gluteus medius pillar and the posterior gluteal line, a thin central segment is present (Fig. 2).

Four dense landmarks are the basis for this ring segment:

- Superior the iliac crest, reaching from the anterior superior iliac spine (ASIS) to the posterior superior iliac spine (PSIS)
- Anterior the thick and strong bone segment from the ASIS to the pelvic brim inferior to the anterior inferior iliac spine (AIIS)
- Inferior the supraacetabular corridor starting at the AIIS and ending at the posterior inferior iliac spine (PIIS) or the PSIS
- Posterior the strong bone between the PIIS and the PSIS

Thinnest area of the iliac fossa was reported to be 0.7–0.8 mm [74].

### Acetabular ring

The acetabular ring consists of the surrounding bone around the facies lunata and can be divided into the anterior wall, the thicker posterior wall, and the superior dome [21]. The acetabular fossa is usually a thin bone segment of 3–4 mm and less thickness in the elderly [15]. Recent analyses reported a central thickness of the acetabular fossa of maximum 1 mm [30, 66, 79].

The acetabular ring is internally rotated relative to the iliac ring structure.

### Obturator ring

The obturator ring is slightly anteriorly and internally rotated in relation to the acetabular ring. The superior part is the so-called infraacetabular corridor, described by Letournel and Culemann [12, 38] and connects the anterior and posterior column; anteriorly are the superior pubis ramus and part of the inferior ramus and the posterior part consists of the ischial tuberosity extending into the true posterior column. A true hole is already present.

For pelvic ring stabilizations, considering these rings, implant placement can be performed in the periphery of the iliac ring (iliac crest area and posterior ilium) and at the supraacetabular bone corridor. Additionally, the superior pubic ramus is an implant region for retrograde ramus screw insertion or vice versa anterior column screw application. The iliosacral screw application is described in detail in another part of this supplement.

## Views for screw application in pelvic ring injuries

The following views are frequently used intraoperatively while stabilizing pelvic ring injuries/fractures:

- Pelvic anterior–posterior view (PAP)
- Pelvic inlet view (PIV)
- Pelvic outlet view (POV)
- Pelvic hyper-inlet view (PhIV)
- Combined obturator oblique outlet view (COOO)
- Combined obturator oblique inlet view (COOI)
- Combined iliac oblique outlet view (CIOO)
- Combined iliac oblique inlet view (CIOI)
- True lateral view (TLV)

### Pelvic anterior–posterior view (PAP)

**Fig. 3:**
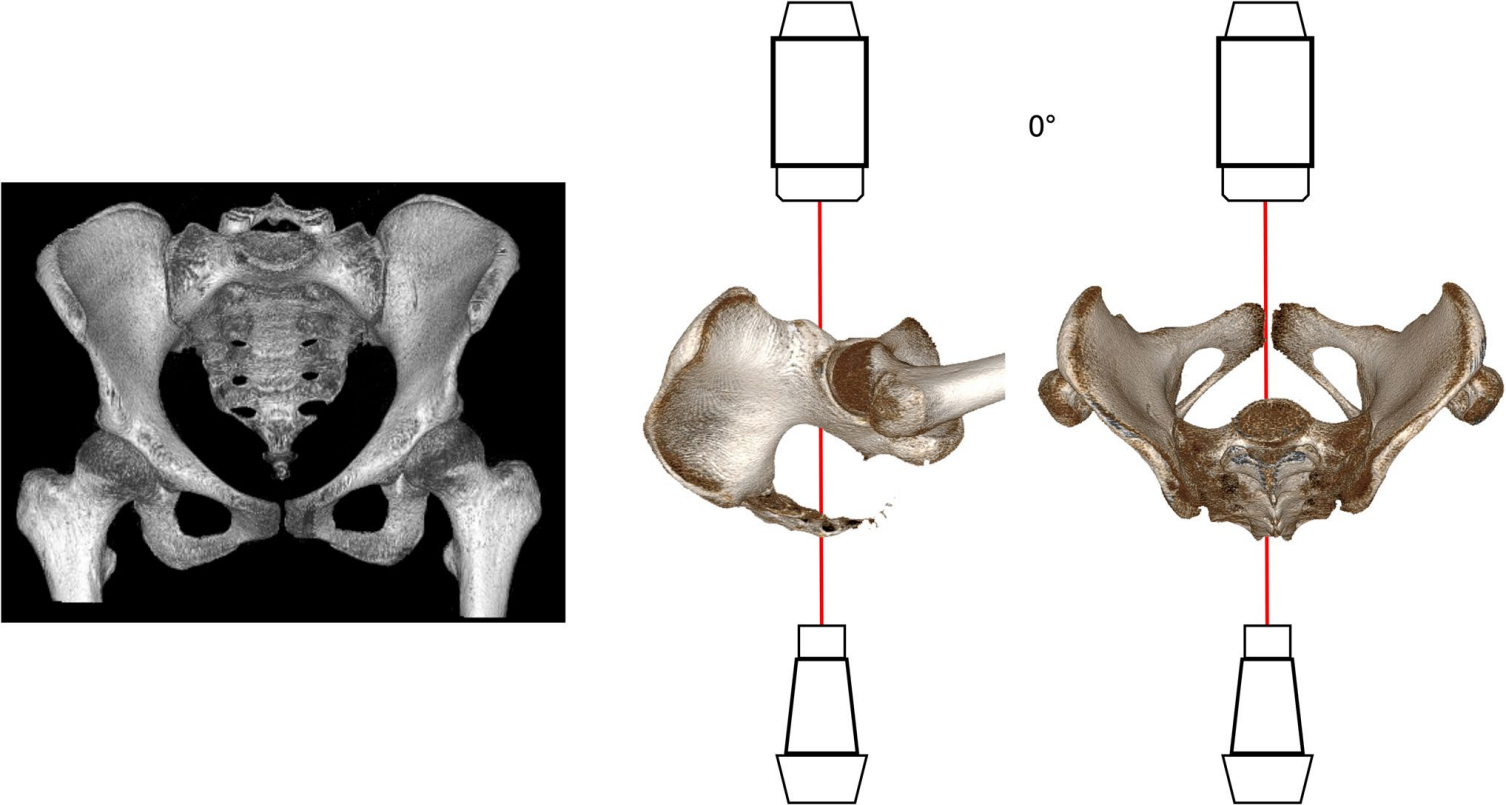
Classical a.p. 3D view of the pelvis with image intensifier positioning

In an optimal PAP both hips are internally rotated by 15–25° from the hip (in profile visualization of both greater trochanters and partial superimposition of the lesser trochanters). The midline of the sacrum should run through the center of the pubic symphysis (Fig. 3). Radiological criteria for an optimal view include visualization of the entire pelvis, symmetrical presentation of the obturator foramina, the acetabular teardrops and the iliac wings. The sacrococcygeal joint projects optimally 1–3 cm superior to upper border of the superior rami [78, 85]. The fracture situation has to be considered.

### Pelvic inlet view (PIV)

Originally, Pennal et al. described this view as being 40° in cranial direction from the PAP [48] (Fig.). Recent analyses revealed an optimal angle of 21–27° at the level of S1 and an angulation of 28° at the level of S2 [47, 51, 86] without influence of gender and age. An overlap of the anterior S1 and S2 bone borders should be achieved.

**Fig. 4:**
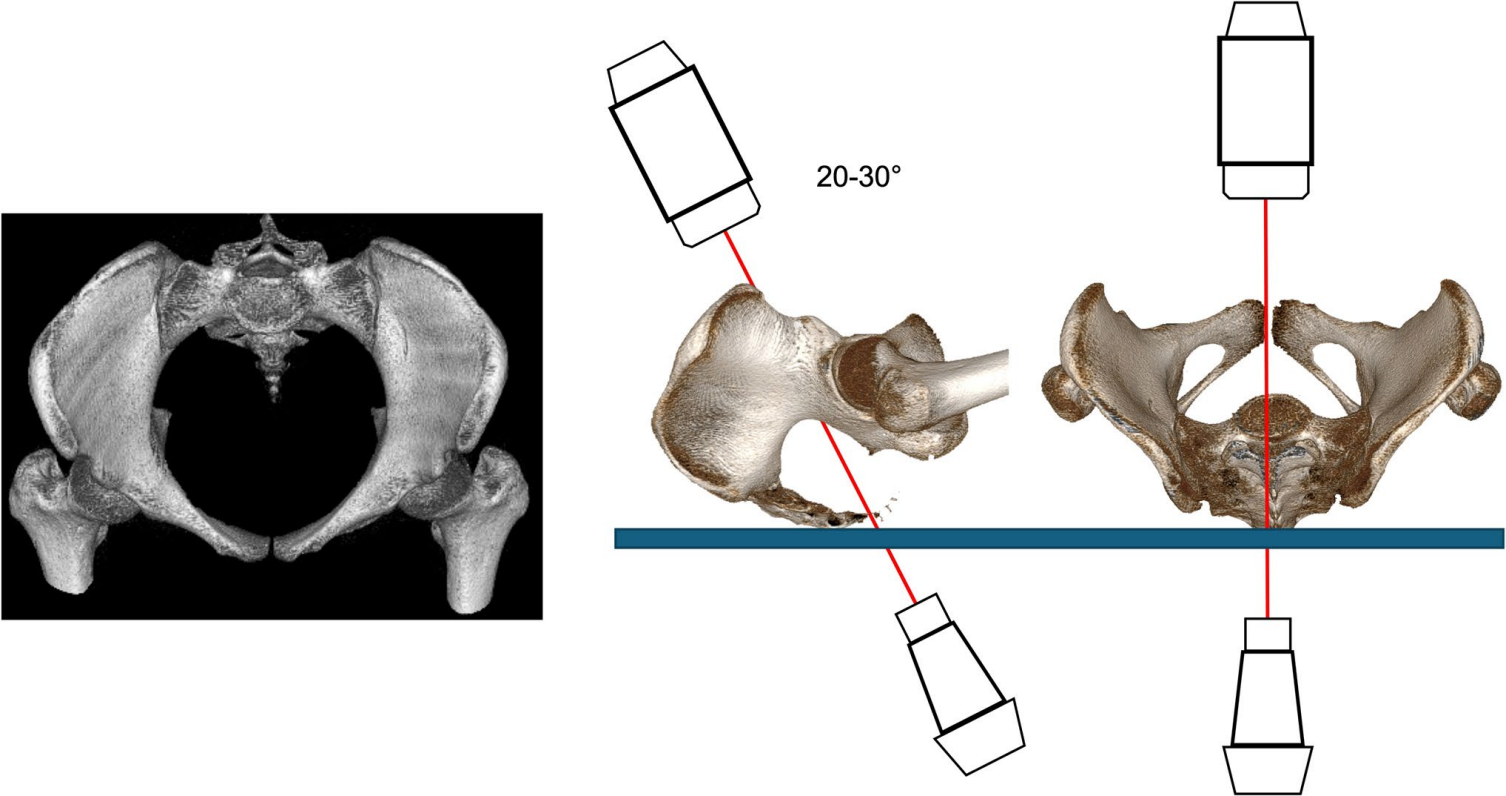
Pelvic Inlet view gained with the image intensifier positioned 20-30° cranially

Thus, the image intensifier is cranially rotated approximately 25° (Fig. 4).

### Pelvic outlet view (POV)

**Fig. 5:**
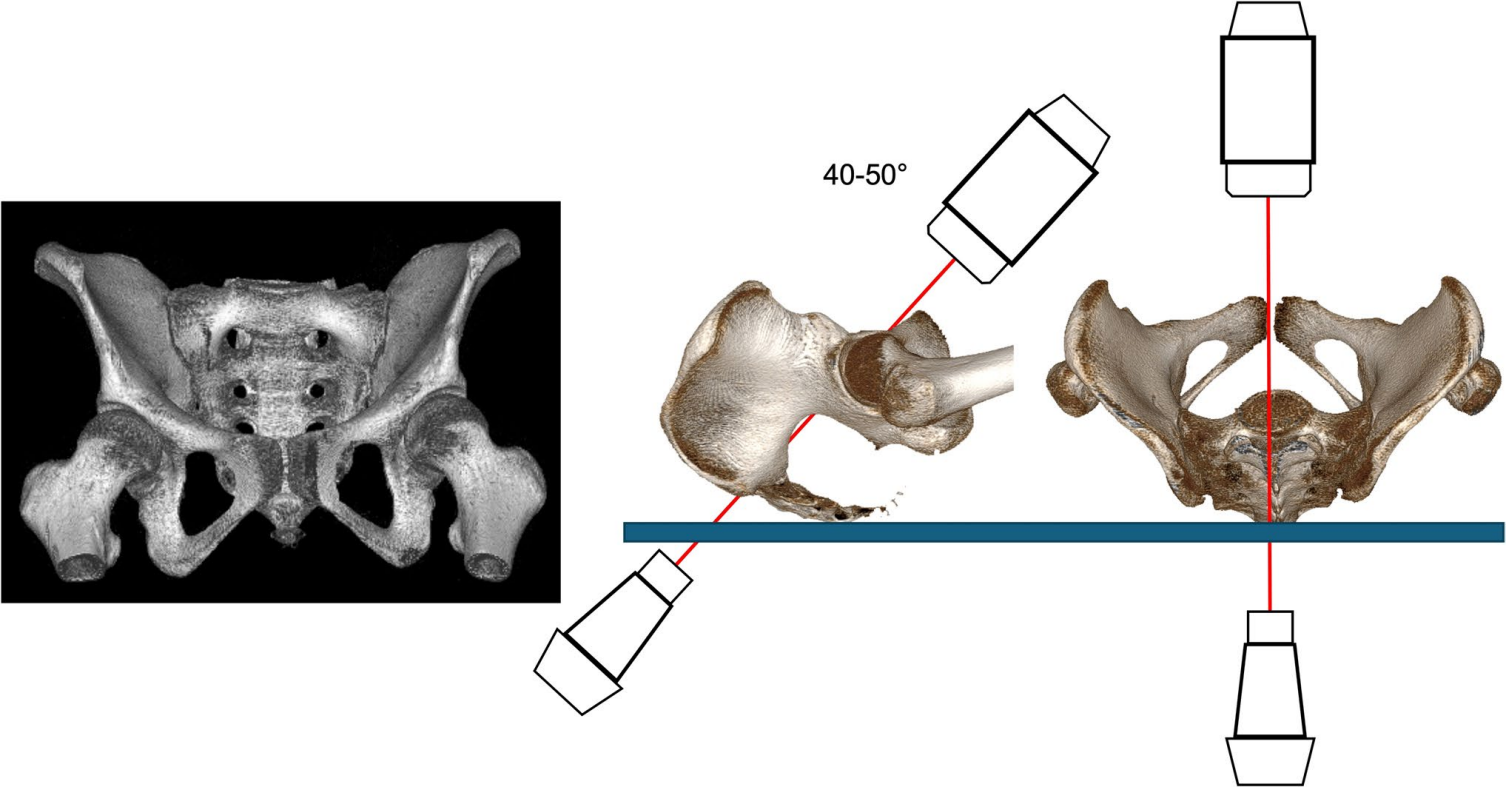
Pelvic Outlet view gained with the image intensifier positioned 50° caudally

Pennal et al. described this view as being 40° in caudal direction from the PAP [48] (Fig. 5). Recent analyses revealed an optimal angle of 43–63° at the level of S1 and an angulation of 52–57° at the level of S2 [47, 51, 86] without influence of gender and age. Sacral dysmorphism lead to an increase of the outlet angle of 5° [47]. Both upper pubic rami lines should be at the level of the S2 neuroforamina.

Thus, the image intensifier has to be caudally rotated approximately 50° (Fig. 5).

### Pelvic hyper-inlet view (PhIV)

The PhIV is used predominantly for retrograde superior ramus screws by creating an overlap of the superior and inferior ramus to allow identification of the starting point at the pubic tubercle. Additionally, the medial and lateral border of the superior ramus can be identified.

**Fig. 6:**
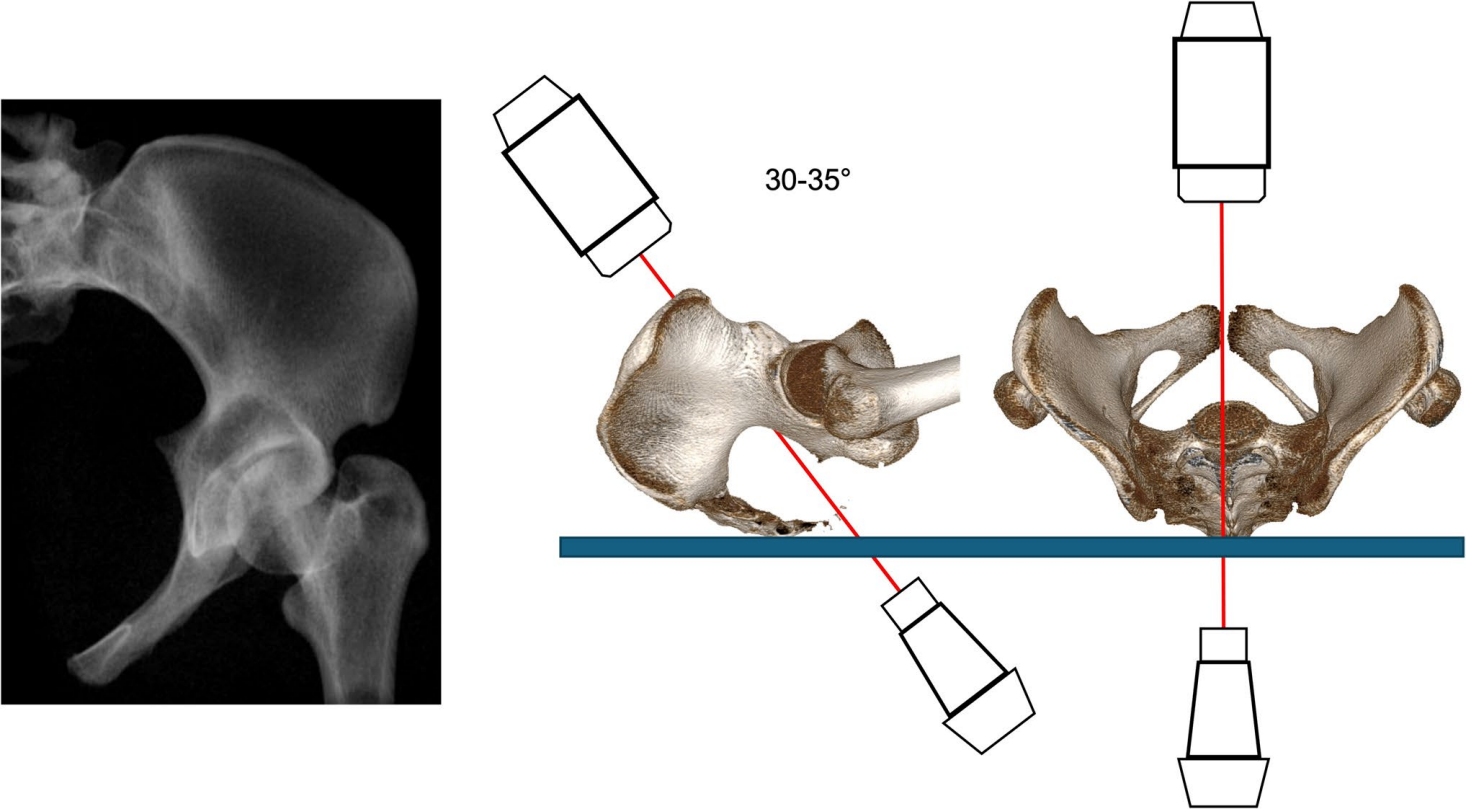
Pelvic Hyper-Inlet view gained with the image intensifier positioned 35° cranially

The image intensifier is cranially rotated approximately 30–35° (Fig. 6).

### Combined obturator oblique outlet view (COOO)

**Fig. 7:**
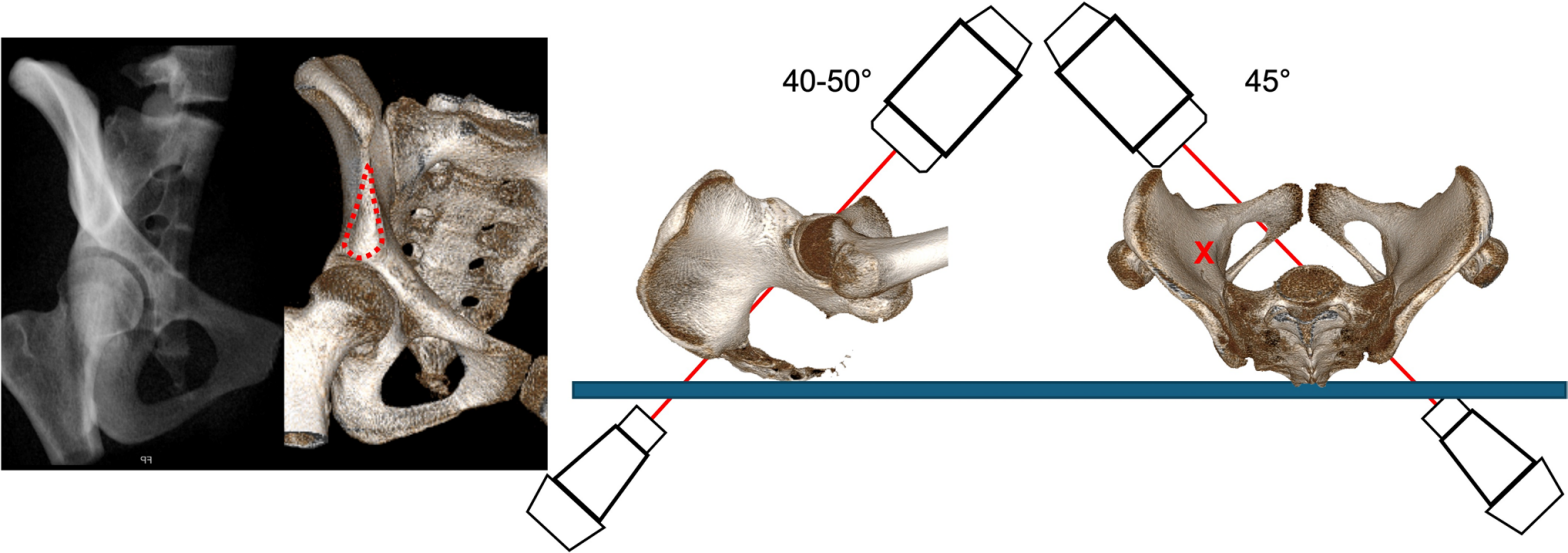
Combined Obturator Oblique Outlet view (COOO). The image intensifier is rotated 50° caudally and 45° lateral to the affected side

The COOO visualizes an axial orientation of the supra-acetabular corridor that runs from AIIS to PSIS, by creating a tear drop figure (Fig. 7, red dots). Additionally, the superior ramus can be observed in full length for application of an anterior column screw or retrograde superior ramus screw. Clinically, the 3D shape of the osseous corridor is visualized two-dimensional.

The image intensifier is usually rotated 45° to the injured side (OOV) and 40–50° caudally (POV) (Fig. 7).

### Combined obturator oblique inlet view (COOI)

The COOI view visualizes the lateral and medial border of the ilium representing the PSIS-AIIS corridor lateral to the SI-joint, which is seen in its maximum shape. Starting with a PAP view the image intensifier is tilted for a PIV view approximately 20–25° cranially until the anterior S1 and S2 cortical lines overlap. This is followed by external rotation of the image intensifier receiver by 40° to an OOV of the anterior ring until the lateral iliac cortex demonstrates a dense line.

The COOI view confirms the periacetabular medial and lateral bone corridor for an ACS. It can also be used for assessment of sacroiliac joint reduction.

**Fig. 8:**
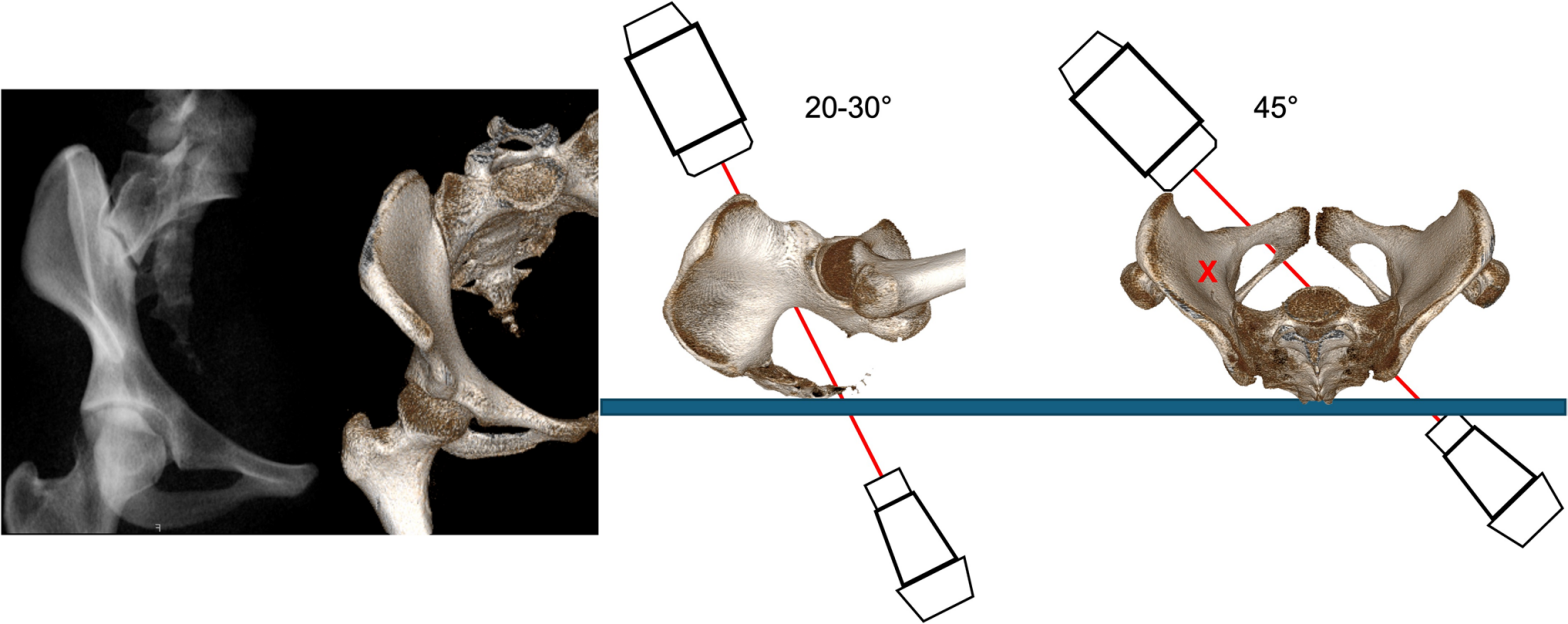
Combined Obturator Oblique Inlet view (COOI). The image intensifier is rotated 20-30° cranially and 45° lateral to the affected side

The image intensifier is usually rotated 45° to the injured side (OOV) and 20–25° cranially (PIV) (Fig. 8).

### Combined iliac oblique outlet view (CIOO)

The CIOO is predominantly used for infraacetabular screw placement and posterior column screw placement [8, 26].

**Fig. 9:**
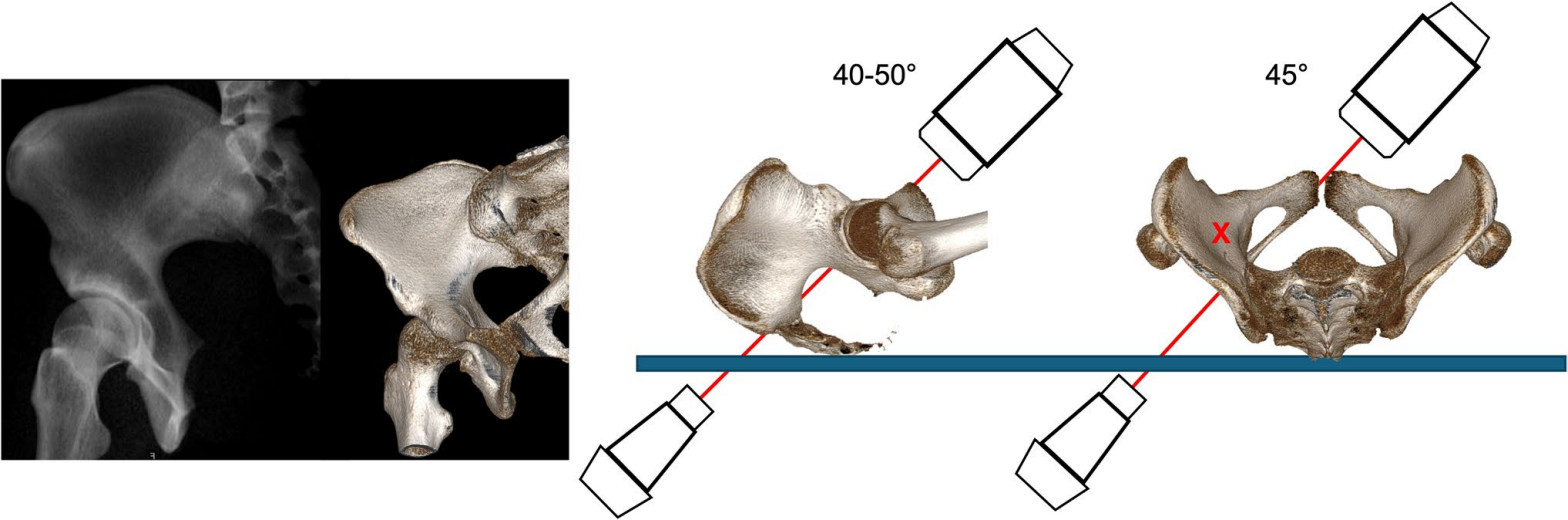
Combined Iliac Oblique Outlet view (CIOO). The image intensifier is rotated 40-50° caudally and 45° medial to the affected side

The image intensifier is usually rotated 45° away from the injured side (IOV) and tilted 40–50° caudally (POV) (Fig. 9).

### Combined iliac oblique inlet view (CIOI)

**Fig. 10:**
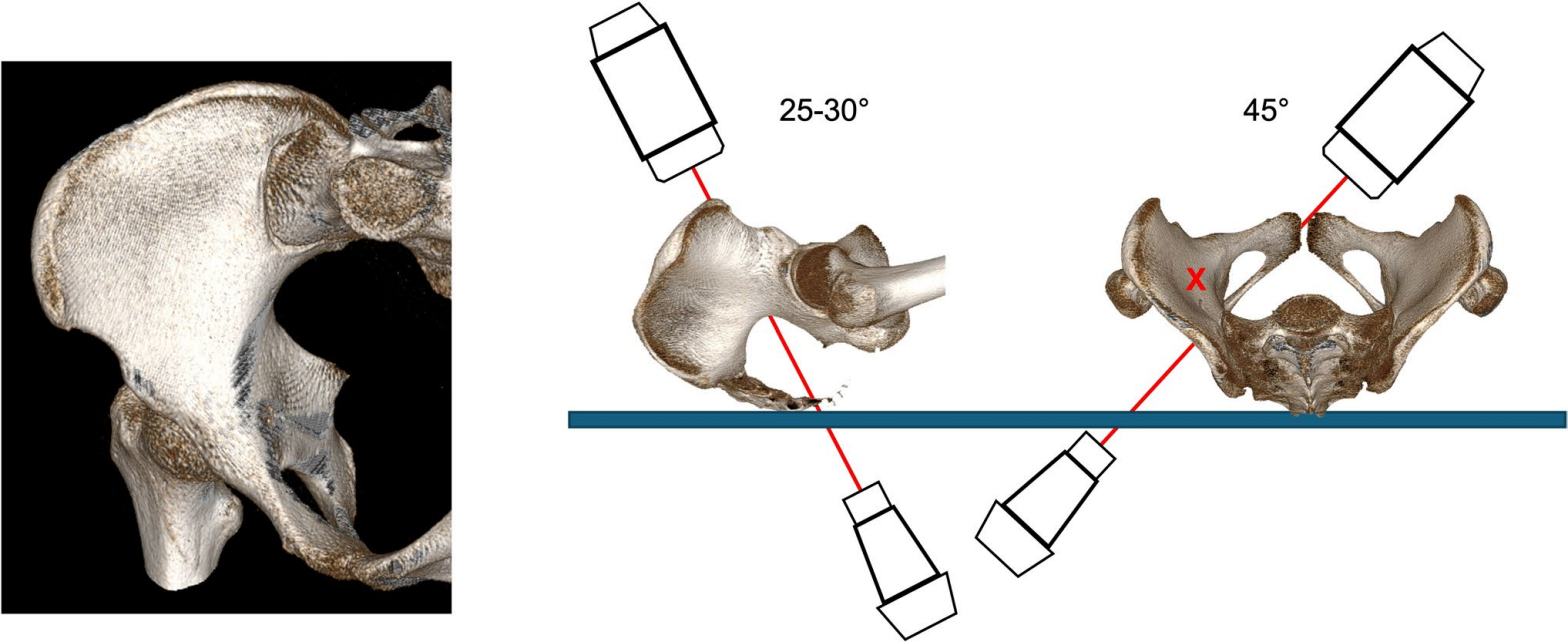
Combined Iliac Oblique Inlet view (CIOI). The image intensifier is rotated 20-30° cranially and 45° medial to the affected side

The CIOI additionally confirms intraosseous course of posterior column screw position [8]. The image intensifier is usually rotated 45° away from the injured side (IOV) and tilted 25° cranially (PIV) (Fig. 10).

### True lateral view view (TLV)

The TLV shows overlapping of both hemipelves. The hemipelvis more close to the x-ray receiver is projected smaller compared to the hemipelvis on the side of the x-ray beamer.

**Fig. 11:**
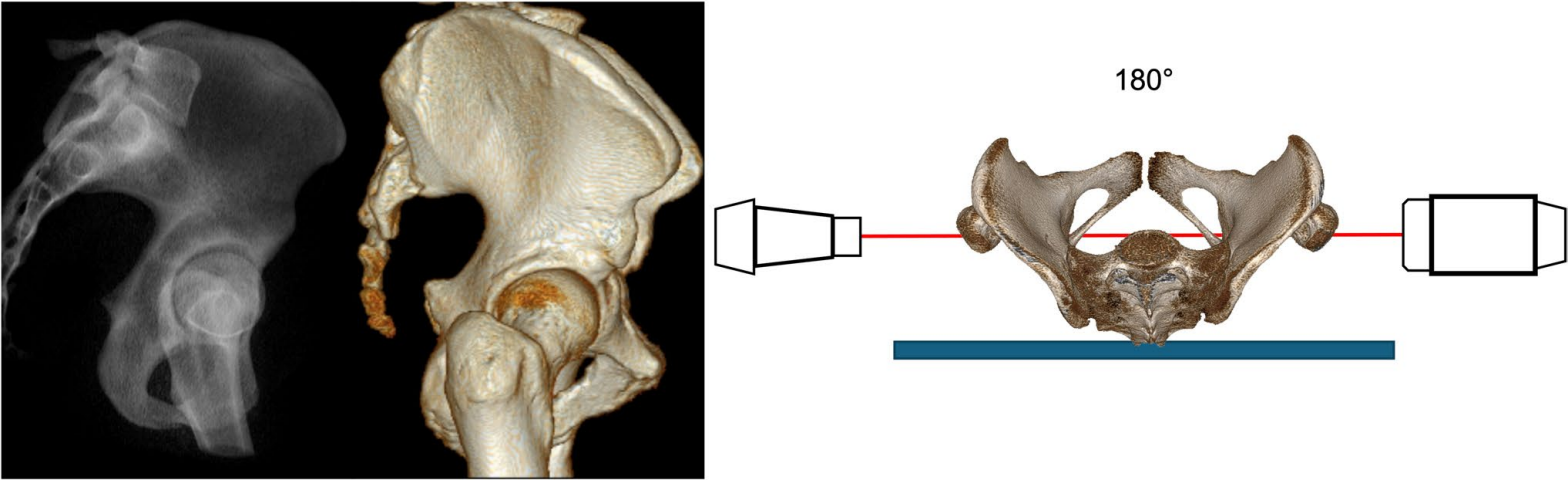
True Lateral View (TLV) with overlapping of both greater sciatic notch lines and the iliac cortical densities

Overlapping of both greater sciatic notch lines and the iliac cortical densities are prerequisites for an optimal view. Additionally, both acetabular circles should be superimposed (Fig. 11).

The following radiographic screw corridors will be described in detail as they are relevant for pelvic ring fixation:

- Superior ramus corridor (SRC): retrograde superior ramus stabilization
- Anterior column corridor (ACC): antegrade superior ramus stabilization
- Supraacetabular corridor (SAC)
- Retrograde (posterior-to-anterior) for lumbopelvic fixation
- Antegrade (anterior-to-posterior) for Lateral Compression 2 screw
- S2-Ala-Iliac corridor (S2AI)
- Modified SAC
- GLUTEUS Medius Pillar Corridor
- Iliac crest corridor (ICC)

## Superior ramus corridor (SRC) for retrograde superior ramus screw application

**Fig. 12:**
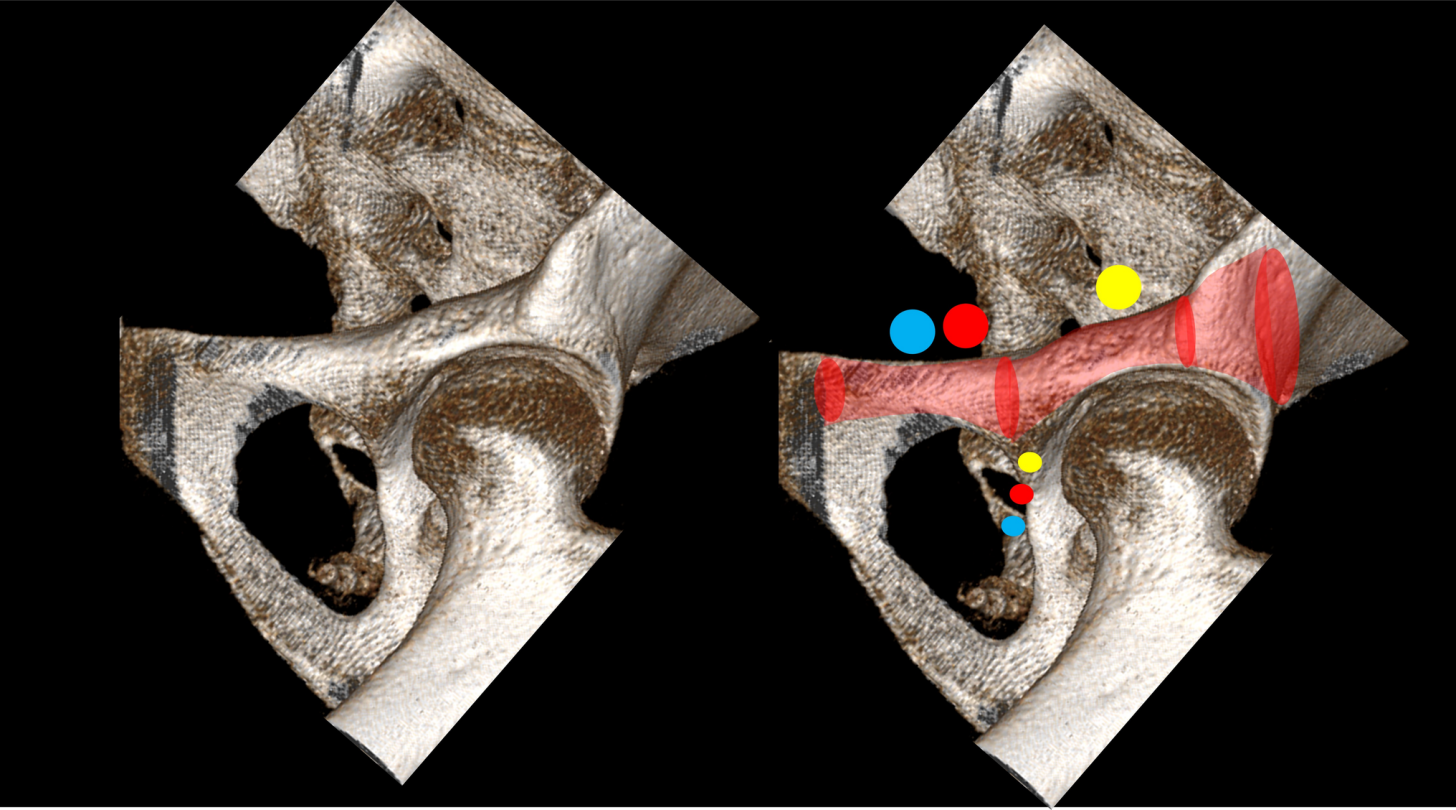
Periosseous anatomy of the superior ramus corridor on the Combined Obturator Oblique Outlet view (COOO)

The superior pubic ramus/anterior column bone corridor is not a linear cylinder. It has more a double golf pie structure (Fig. 12). Application of a retrograde screw into this corridor was already described by Lambotte in 1913 [36]. The osseous corridor starts close to the pubic symphysis and contains the entire upper pubic rami up to the anterior acetabular border, the anterior wall, and ends in the area of the pelvic brim near the SI joint.

Relevant surrounding anatomical structures include the corona mortis, the obturator canal with the obturator neurovascular bundle, and the iliac external vessels. The superior border is extremely variable due to several bony landmarks [82]. After few centimeters from the pubic tubercle the iliac external vessels groove represents a first narrow zone, followed by the prominent iliopectineal eminence opposite to the anterior horn of the acetabulum. Lateral and posterior to the iliopectineal eminence, the iliopsoas groove (iliopsoas gutter) is the second narrow zone, which ends at the level of the AIIS.

The narrowest region is observed between the iliopsoas gutter and obturator neurovascular canal [2, 59]. The clinically most relevant isthmus is the superior distance between the joint and the superior corridor border. The reported measurements differ depending on the chosen measurement location [3, 10, 49, 50, 68].

The medial part of the SRC has a curved shape, consisting of different curvature values [3], corresponding to the iliopectineal line. The inferior border of the SRC is irregular, starting with a triangle- shaped configuration at the upper pubic ramus extending into the obturator canal and the anterior horn and wall area. There is an ongoing change in cross-sectional anatomy of the SRC [4, 34]. At the upper pubic rami there is a change from triangular to circular [4, 34]. Around the joint area, the circular orientation changes to horizontal ovoid to superior-based small triangular at the mid anterior acetabulum to an acetabulum- based triangular configuration at the posterior part of the corridor until an oblique rhombus-like configuration posterior to the acetabulum [34].

### Retrograde screw starting point

SRC screws are indicated in mid superior ramus fractures or fractures just cranial/lateral to the obturator foramen [63].

The distance between the center of the pubic symphysis and the entry point was found with an average of 27–28 mm [16], while the distance between the retrograde screw entry point and the mid symphysis was 18.4 ± 4.8 mm [10]. The distance between the entry point and the pubic tubercle was 14–17 mm 51 and the distance from the superior rim of the upper pubic ramus was 17.8 ± 2.6 mm [10].

**Fig. 13:**
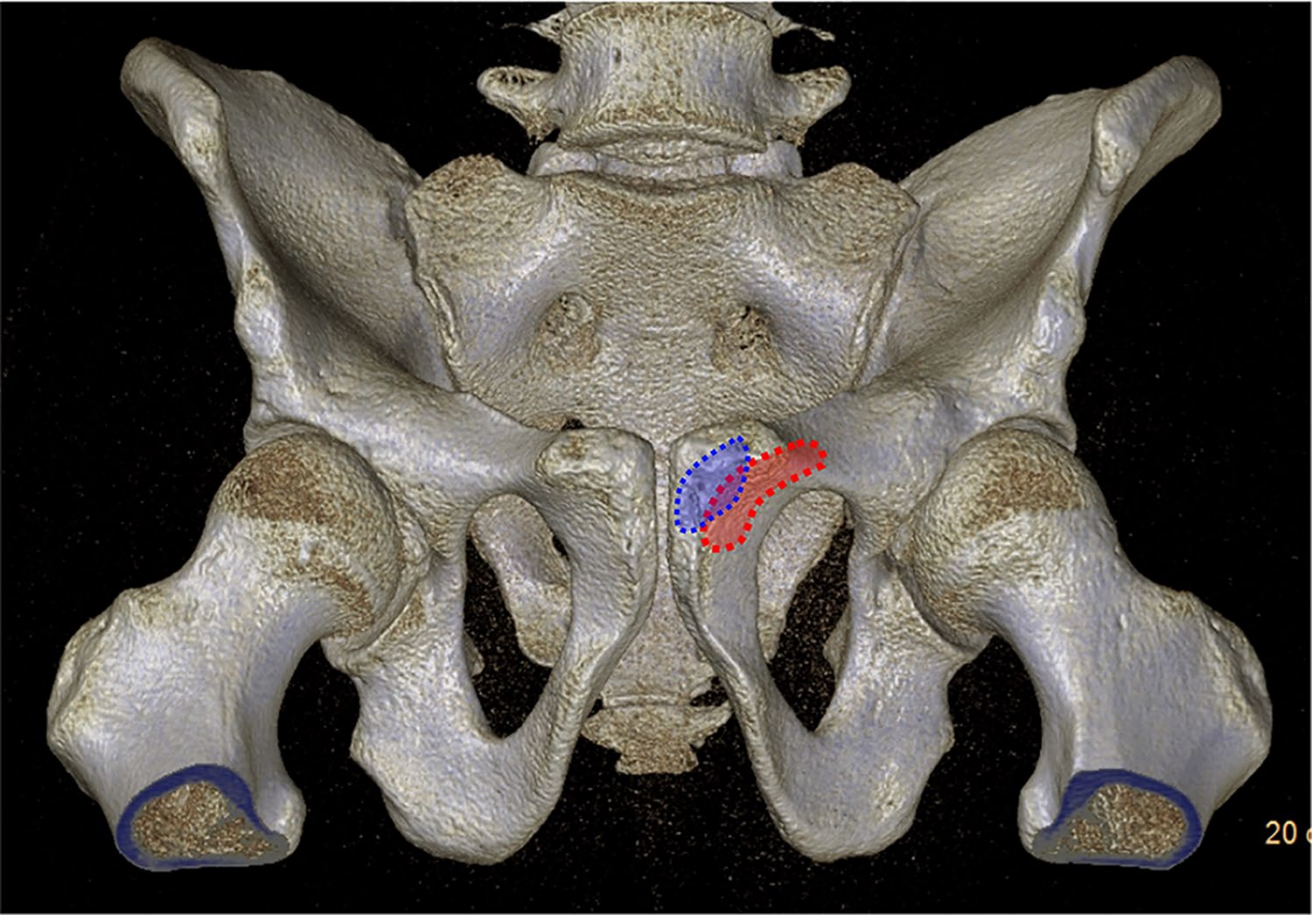
Crescent shaped starting area at the symphyseal region for retrograde screw application (males (blue), females (red))

Depuis et al. could not identify a universally reproducible entry. A crescent shaped area different between males and females (more lateral) was reported [17] (Fig. 13).

**Table:** 

The appropriate osseous start point is just beneath the pubic tubercle	

### Guide wire/screw angles

A CT analysis in Japanese people revealed a screw angle of 54.1° in males and 55.9° in females in lateral direction to the sagittal plane and 66° and 67° in the vertical direction relative to the horizontal plane [68].

### Radiographic views

Radiographically, views are optimal if they can identify the cranial–posterior cortical bone (the pelvic brim), the superior acetabular subchondral bone (acetabular dome), the mid-ramus caudal bone, and the flattened curved posterior cortical surfaces [18]. Eastman et al. recommended the PIV and the COOO. No standard values for image intensifier positioning were reported due to individual and gender anatomy. No full superimposition of the superior and inferior pubic ramus (PhIV) was recommended [18]. Using the PIV, the posterior superior ramus surface is located just posterior to posterior inferior ramus surface (identification of a small obturator foraminal shadow). The mid ramus area is insufficiently visualized with this view.

The COOO view is optimal, if it shows the maximum width of the mid ramus area which requires some fine-tuning of the image intensifier.

Rommens et al. additionally recommend the PAP. The PIV shows a view from “above”, the COOO view from lateral [53].

**Fig. 14:**
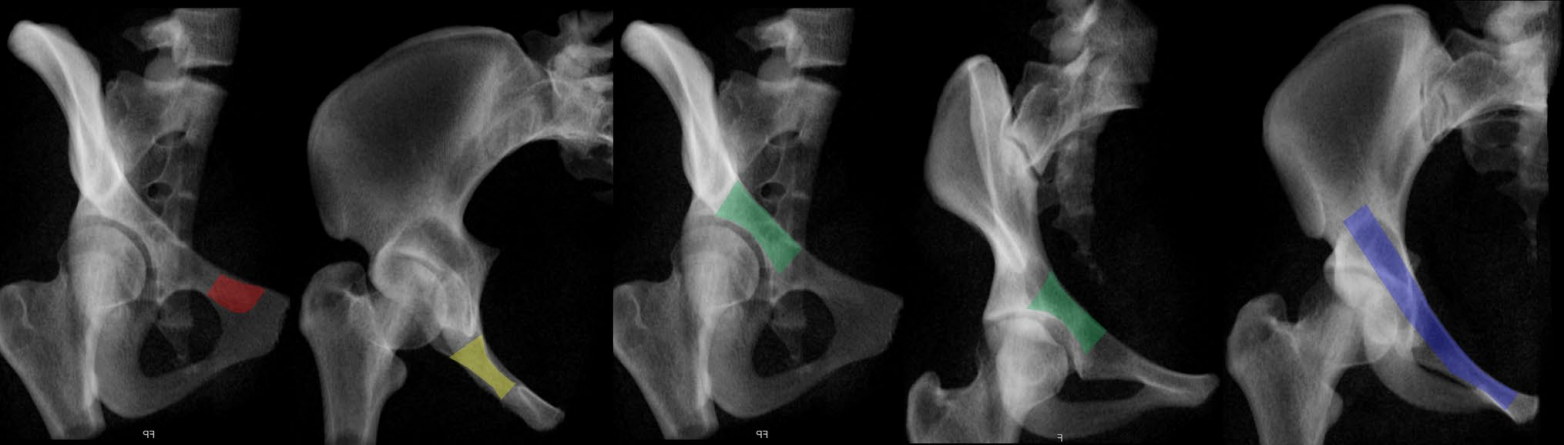
Radiographic sequence for retrograde superior ramus screw or antegrade anterior column screw: COOO, hyper-Inlet, COOO, COOI and pelvic Inlet view with corresponding areas for optimal guide-wire analysis (colored)

The recommended radiographic sequence consists of (Fig. 14):

- COOO to identify the entry point close to pubic tubercle
- (Superimposed) PIV until the drill bit tip is close to the (classical) fracture line
- COOO for confirmation of the drill orientation periarticular (an additional COOI view is helpful to have a second plane)
- PIV to analyze the complete course within the superior ramus

In a recent review, the COOO and PIV were favored for anterior column screws and retrograde ramus screw application [32].

The COOI view can be additionally used for confirming the periacetabular medial and lateral bone corridor. Medial, intrapelvic crew penetration can be excluded.

Biomechanically, no difference was found between insertion of two small fragment screws or one 7.3 mm screw [1].

In a forensic CT analysis, a gender difference of the superior ramus bone corridor was confirmed, with a larger corridor in males compared to females (average 8.9 vs. 7.1 mm) [14]. The average length of this corridor was 123 vs.125 mm, respectively.

## Anterior column corridor (ACC) for antegrade anterior column screw (ACS) application

The ACC differs in terms of screw indications. It is often used in acetabular fracture stabilization, especially in fractures with a transverse fracture component to address and fix the invisible contralateral column using the Kocher-Langenbeck approach. For pelvic ring stabilization this approach can be used to antegrade stabilization of anterior superior ramus fractures.

### Entry point

The optimal entry point is clinically difficult to find. The skin incision is recommended at the crossing point of two lines: the lateral femoral border junction line through the greater trochanter and a tangential line from the pubic tubercle symphysis to the AIIS on a pelvic AP view [5].

The optimal entry point for the antegrade ACS at the outer iliac bone was historically described by several authors [19, 38, 68, 83]. According to Letournel, the starting area is on the outer side of the ilium approximately 3–4 cm cranial to highest point of the acetabulum slightly posterior to the anterior gluteal line in a 2 cm circle [38]. Ebraheim et al. described the entry point as 46 ± 6 mm superior to the superior acetabular rim, and 16 mm superior to the midpoint of a line connecting the apex of the greater sciatic notch and the mid-distance between the ASIS and AIIS; sagittal angulation of 90.6° and transversal angulation of 29° were stated [19]. Yi et al. described the entry point as being “slightly superior to the acetabulum in the gluteus medius pillar, a thickening of bone that runs from the acetabulum to the iliac crest.” [83]. The insertion point landmark for an antegrade anterior column screw was found at the intersection between two perpendicular lines, one from the tip of the anterior inferior iliac spine and from the superior edge of the acetabulum [68]. Depending on the size of the bony corridor, antegrade insertion had a possible area around the optimal entry point of 2.5cm^2^ in patients with an 8.0 mm canal diameter and of 5.7cm^2^ in patients with a 14.0 mm canal diameter. The distance between the entry point for an antegrade anterior column screw and the apex of the greater sciatic notch was 37–42 mm [15].

**Table:** 

All these descriptions are theoretical as they cannot be adequately visualized intraoperatively while performing a percutaneous approach	

Eastman described the entry point to be “typically several centimeters cranial to the acetabular dome in the supra-acetabular region near the base of the gluteus medius pillar” [18].

Bozzio et al. used a conventional ap x-ray and defined the starting point of the ACS at the junction of a line from lateral border of the femur shaft axis and a line from the pubic tubercle to the inferior border of the AIIS. The obturator oblique and inlet view are used for confirmation of the potential screw path using a K-wire [5].

The starting point is slightly superior to the acetabulum at the gluteus medius pillar [83].

The starting point on the COOO is approximately 1–2 cm cranial to the lateral-superior border of the superior acetabular rim (outlet–sourcil).

### Length of the ACC

Several authors reported on CT-based measurements of the length of the ACC for antegrade screw placement. Depending on their definition of this corridor, different results were reported. A mean length is approximately 120 mm [10, 49, 50, 59, 68].

### Guide wire/screw angles

The screw orientation is of clinical relevance. Therefore, beside fluoroscopy, intraoperative guidance is helpful for screw insertion and angulations of the drill bit can help to determine the optimal screw course:

- A lateral-superior to medial-inferior directed angle of 33.6–39° on the PAP [10, 49, 50, 88]
- A posterosuperior to anteroinferior directed angle of 15–22.3° on the TLV [49, 50, 68]
- A posteromedial to anterolateral directed supraacetabular angle of 55–59.1° in the horizontal/transverse plane (inlet view) [10, 68, 88]

### Radiographic views

Interestingly, compared to retrograde screws, all possible standard views and their combinations were recommended including the standard PAP, OOV, IOV, COOO, PIV, COOI, CIOI, and CIOO [25–27, 43, 52, 64, 67].

Beside the classical PIV and POV, the COOO seems to be relevant. Further views were reported recently, and combinations of these different views are potentially the most helpful intraoperative tool [83].

Practically, sequential views are optimal for intraosseous guide wire confirmation along the ACC. After identification of the starting point using the COOO, the lateral and central guide wire course is monitored using the IOIV or PIV. The COOO is used exclude hip joint penetration. IOIV and PIV can exclude medial ramus penetration [64, 81].

Disagreement exists whether an overlapping of the superior and inferior pubic rami, forming one column, is helpful using the PIV [72, 80] or should be avoided [4]. This overlapping view was described by Yi as an CIOI [83]. The guidewire should be targeted to the midline of the upper pubic rami from posterior (antegrade orientation).

The COOO confirms avoidance of hip joint penetration and of a superior extraosseous pathway. Outlet configuration of this view should be extended to the maximum width of the bone corridor between the joint and the iliopectineal eminence. Due to the upper pubic rami slope (vascular groove) [4], the guidewire should be target to the superoposterior (most proximal) cortex.

The COOI view confirms the periacetabular medial and lateral bone corridor. Medial, intrapelvic crew penetration can be excluded.

**Table:** 

The COOO and the PIV are the dominantly used intraoperative images [32]	

In an experimental analysis of implantation of an ACS using five distinct trajectories, standard radiographic views were performed which were presented to 32 pelvic and acetabular surgeons [28]. The COOO view was rated optimal in terms of exclusion of hip joint penetration, while the PIV and the CIOO were optimal for confirming the intraosseous screw course.

In contrast, Wang et al. proposed more the OOV and PhIV. They compared the value of the COOO and inlet view with OO and pubic ramus inlet views [77]. The screw quality (complete intraosseous screw course) was significantly superior using the OO and PhIV (85.5% vs. 58.2%). Even, small superior ramus penetration (< 50% of anterior column) was observed in 7.3% vs. 18.2%, respectively. Also, a larger distance between the screw and the acetabular cavity was observed using the OO and the PhIV (2.78 mm vs. 0.92 mm).

Technically, after confirming the entry point for ACS application and insertion of the guide wire for 2-3 mm, it is withdrawn and inserted again reversed for further blunt hammering [70].

Several new images were reported recently.

### Axial anterior column screw view

**Fig. 15:**
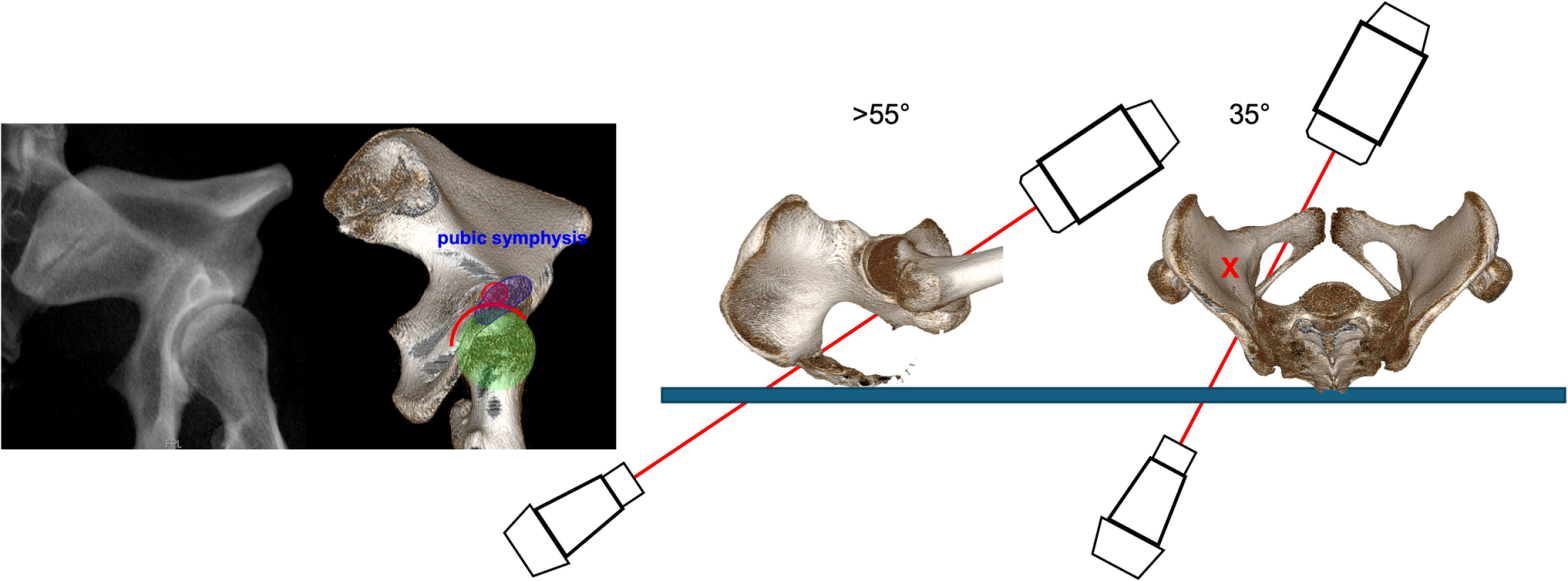
Axial Anterior Column Screw View. The image intensifier is rotated >55° caudally (hyper Outlet) and approx. 35° medial to the affected side

Recently, a new intraoperative image view was described for analysis of sufficient screw position in anterior column screws, the AACS (Fig. 15).

The image intensifier is placed on the healthy side. Starting with an IOV (C-arm is tilted approximately 35° toward the contralateral hip joint), the C-arm is then rotated toward the feet, creating an extended outlet image until an oval track image gradually appears (acetabular anterior column view) [88] The starting point for the guidewire should be centrally placed in this corridor [87]. The typical shape of the superimposed corridor is quadrangular (Fig. 15), but a high variability is observed. A mallet technique is recommended for insertion of the guide pin.

This bony corridor consists of four walls. The superior wall of the corridor consists of the grooves anterior and posterior to the iliopectineal eminence, whereas the inferior border is the direct supraacetabular plane and the bottom of the upper pubic rami. The medial and lateral walls are the sides of the upper pubic ramus.

The ideal guidewire or drill angulation angle is 70–85° in men and 75–90° degrees in women relative to the transverse plane, and 30–40° degrees in men and 35–45° degrees in women relative to the coronal plane [87].

After placing the guidewire or drill, position is confirmed additionally using inlet and outlet views [87].

### Modified CIOO

**Fig. 16:**
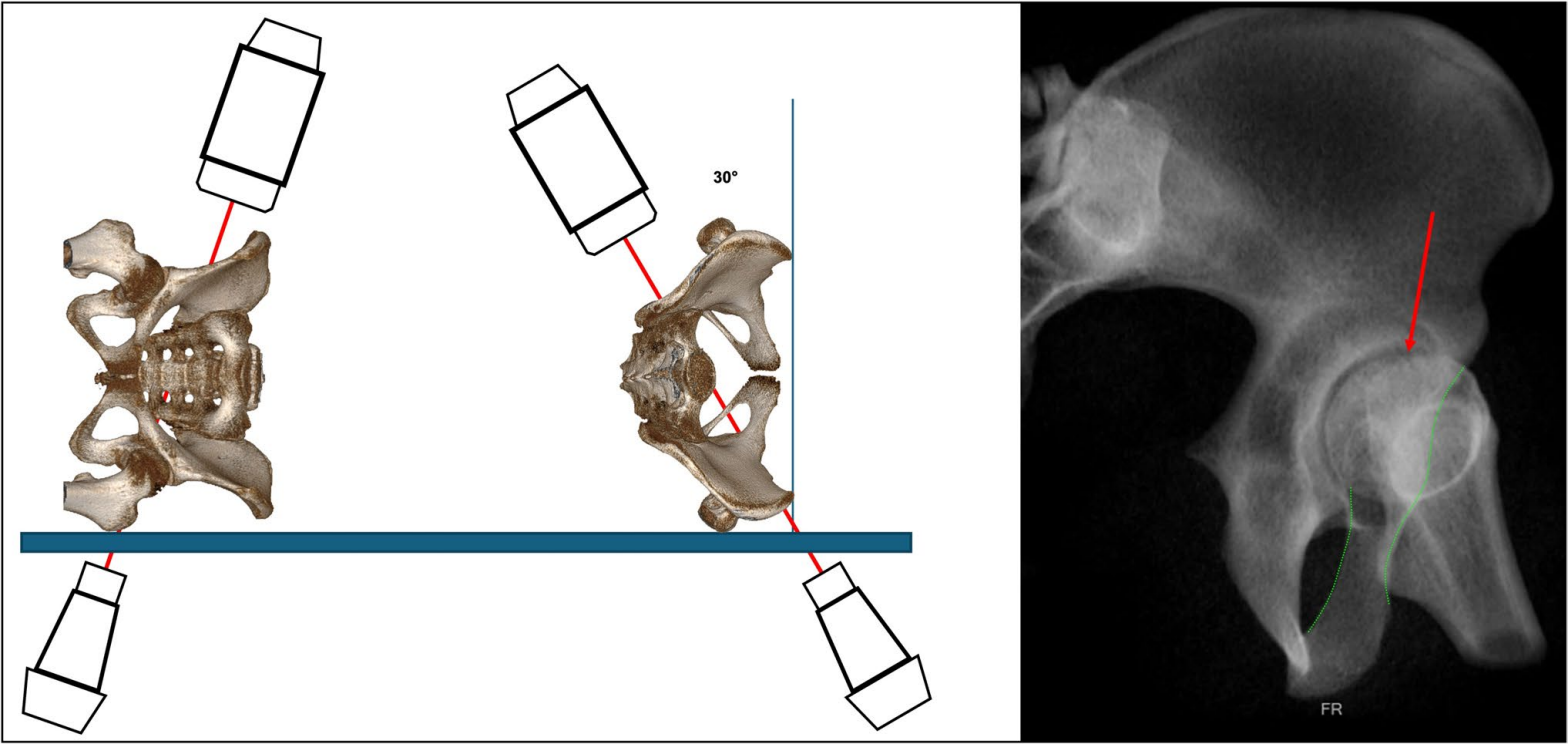
Modified Combined Iliac Oblique Outlet view (mCIOO). The image intensifier position starts with a 30° outlet position in the lateral decubitus position of the patient, then rotated approximately 50° to the vertical plane to get the COOO view. Further 80-degree rotation in the C-arm plane results in the mCIOO view

Cunningham described the modified iliac oblique outlet view (mCIOO) as a coplanar view (very near orthogonal) to the COOO that allows direct visualization of the upper pubic ramus and the pubic tubercle for easier identification of the antegrade anterior column screw starting point [13]. In the lateral decubitus position of the patient, starting with a 30° outlet position of the C-arm, first the C-arm is rotated approximately 50° to the vertical plane to get the COOO view. Further 80-degree rotation in the C-arm plane results in the mCIOO view (Fig. 16).

As an option, the optimal intraosseous pathway can be confirmed by careful radiopaque contrast medium application into the screw corridor [72].

## Supraacetabular corridor (SAC)

The supraacetabular screw corridor is a frequently used corridor in pelvic ring stabilization. Typical indications for applying screws include antegrade pin placement in supraacetabular external fixation [23, 40], retrograde screw placement in lumbopelvic fixations [57, 83], S2 alar-iliac (S2AI) screw application, a modification of the classical posterior fixation technique, starting at the posterior sacrum between the S1 and S2 neuroforamina traversing the SI-joint and following the supraacetabular bone corridor, resulting in advanced strength especially in osteoporotic bone [9, 62] and for stabilization of crescent fractures of the pelvic ring [39, 60, 65].

Recently, screws inserted into this corridor were described as LC-II screws [65] according to the fracture classification of Young-Burgess which includes a LC-II subtype to stabilize complete posterior ilium fractures and crescent fractures.

Another indication are fragility fractures type IIIA according to Rommens [44] or acetabular fractures with a high anterior column fracture can be stabilized using this technique [38].

**Fig. 17:**
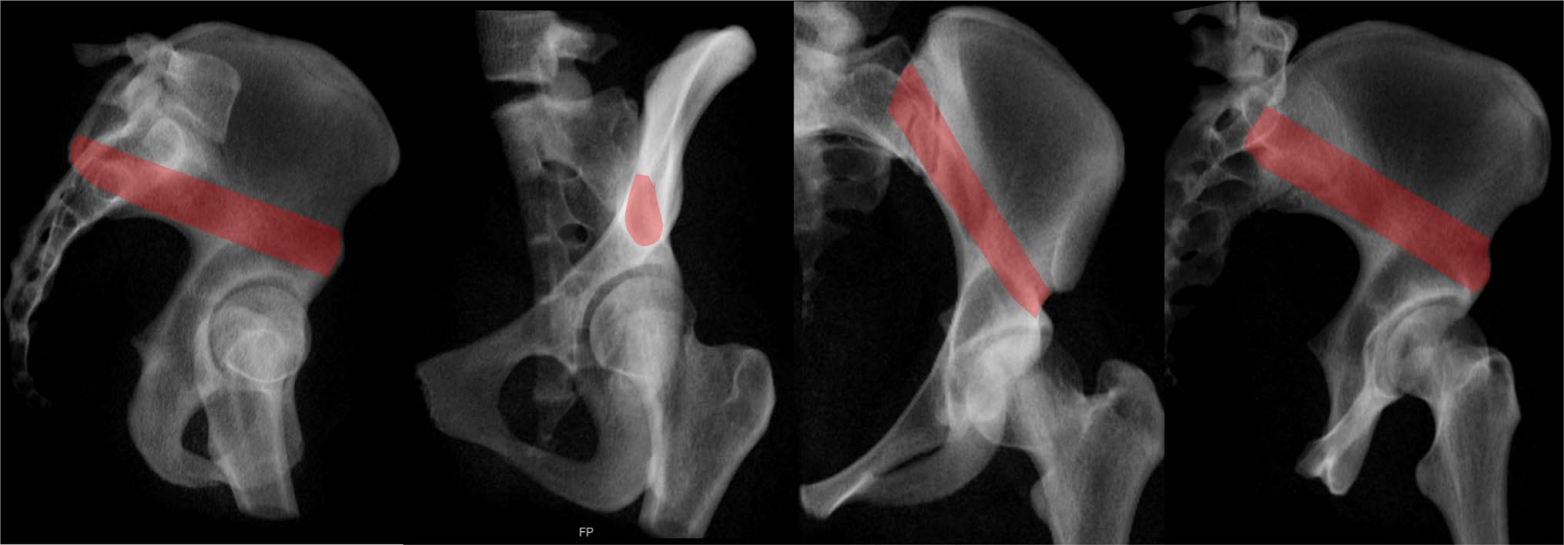
Radiographic visualization of the supraacetabular corridor (SAC) using the true lateral view, COOO, pelvic Inlet and CIOO views (from left to right)

The bone corridor starts at the Anterior Inferior Iliac Spine (AIIS), runs parallel and slightly lateral to the pelvic brim, and superior to the greater sciatic notch before reaching the PSIS or the PIIS (Fig. 17). Thus, the borders of this corridor are laterally the outer iliac surfaces, medially the inner iliac surfaces, superior the thinning between both cortices, and inferior the anterosuperior acetabulum, the greater sciatic notch with the iliac part of sacroiliac (SI) joint.

In males, there are two narrow zones (anterior to and posterior to the gluteal pillar), while in women three constriction points were observed (anterior to and posterior to the gluteal pillar and at the level of the SI joint [75].

### SAC length

Schildhauer defined the overall pathway and two narrow zones of the SAC [57]. The PSIS–AIIS length was on average 141.1 mm in male patients and 128.7 mm in female patients [57]. Comparable lengths were reported by Pichler et al. with 148 mm [49, 50], while De Bondt et al., found no relevant gender difference (males 14,0 mm vs. females 14,5 mm) [14]. The PIIS–AIIS length was on average 86.3 mm in male patients and 99.7 mm in female patients, respectively [57].

**Fig. 18:**
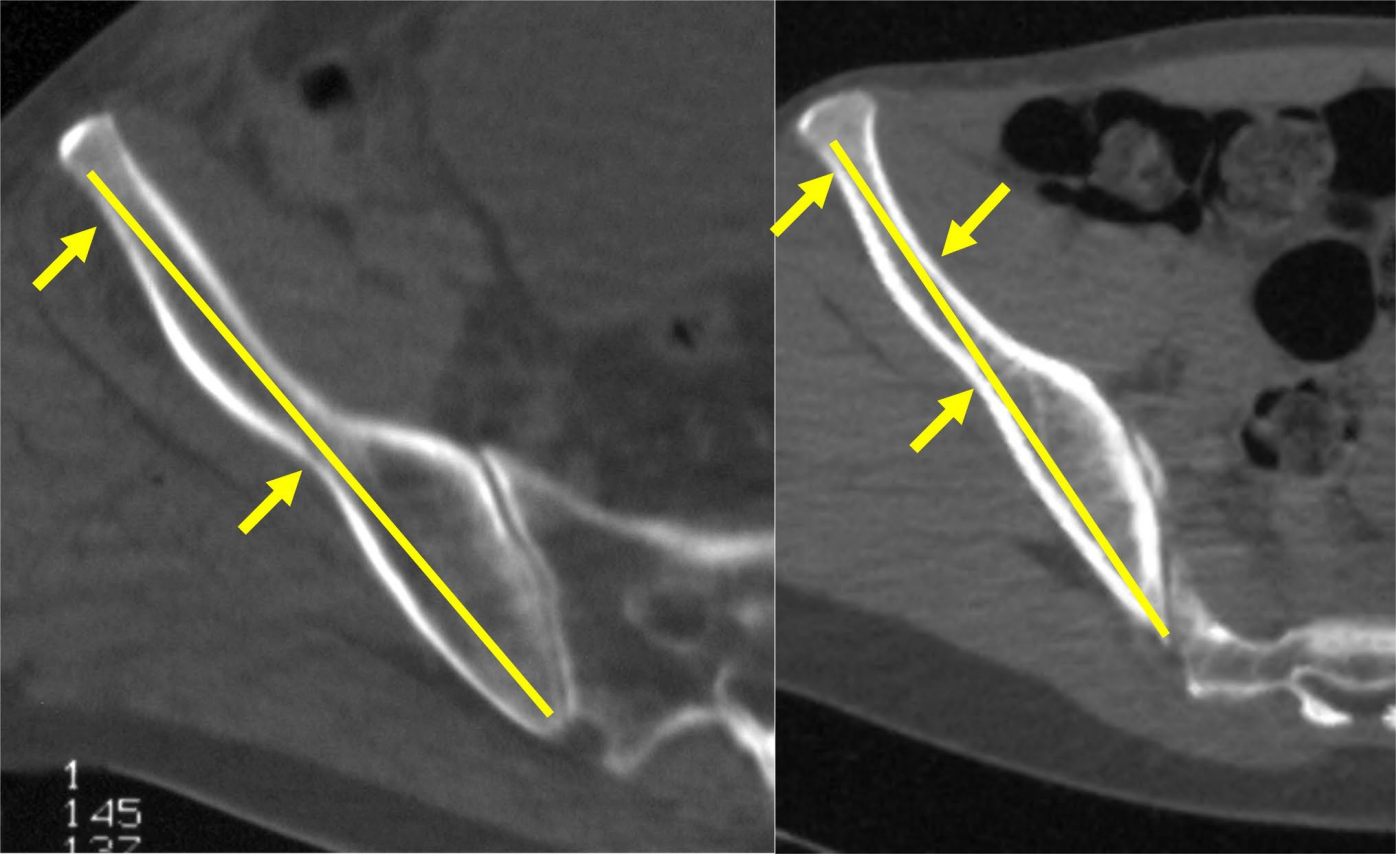
Narrow zones of the supraacetabular corridor (SAC) in axial CT-scans

Two narrow zones are present within this corridor (Fig. 18). The distance to the first narrow area starting from posterior was approximately 3 cm in men and 2.7 cm in women [57]. The distance to the second narrow area was 86.3 mm in men and 84.1 mm in women on the superior pathway (PSIS–AIIS) and 60.3 mm in men and 52.8 mm in women on the inferior pathway (PIIS–AIIS), respectively. Thus, a three-point stabilization of a long screw was considered possible in this corridor [57].

**Fig. 19:**
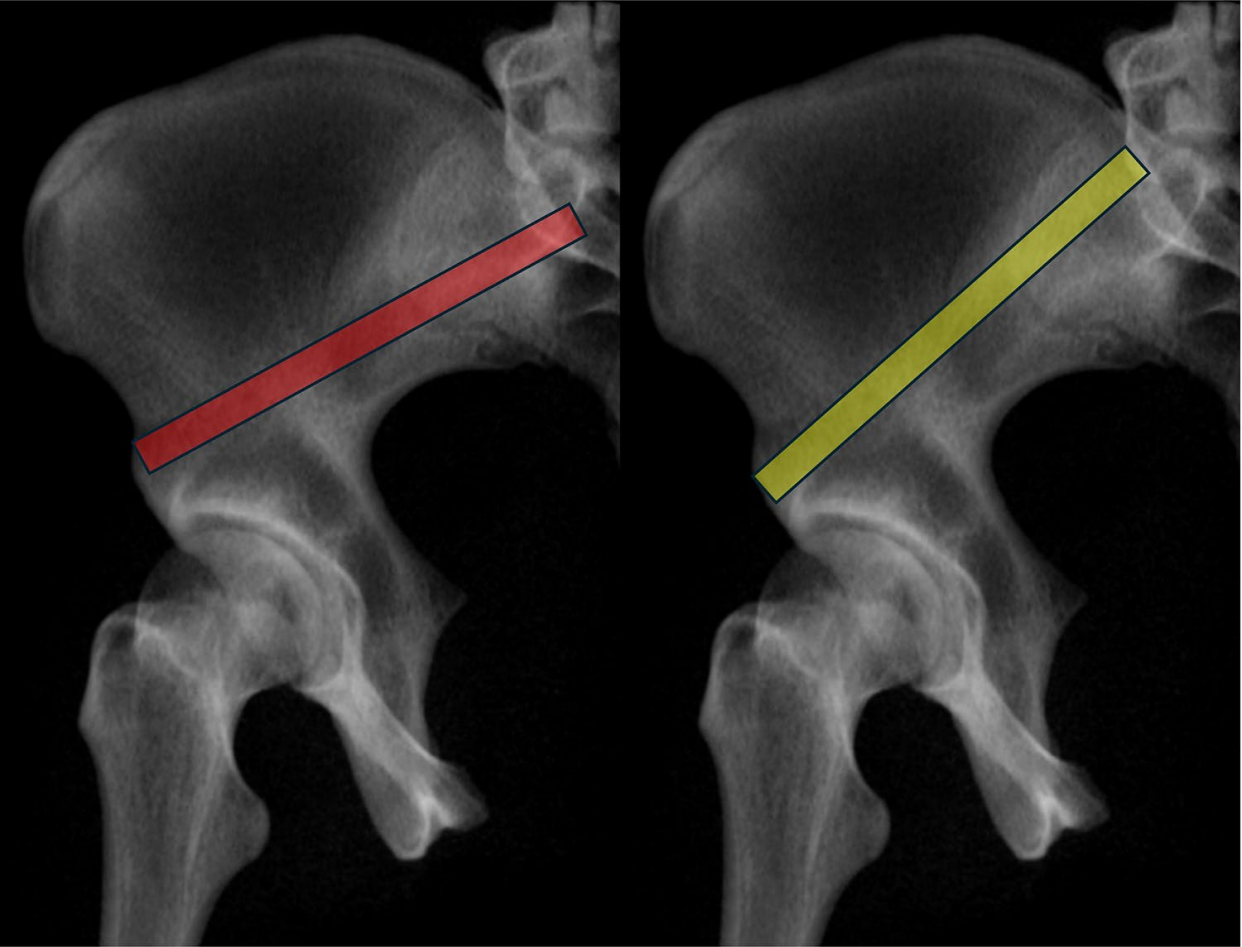
Alternative supraacetabular corridor (SAC) with a more caudal starting point (caudal to the AIIS) and a more cranial angulation (yellow) compared to the standard corridor (red)

The mean diameter of the SAC is 8.3 mm in men and 6.2 mm in women. This difference is due to a more S-shaped ilium in women. A larger alternative SAC measured 11.3 mm in men and 9.9 mm in women. The alternative SAC has a more caudal starting point (caudal to the AIIS) and a posterior exit point cranial to the PSIS (Fig. 19), resulting in a more cranial angulation [75].

This alternative SAC includes the risk of intraarticular starting, as the hip joint capsule insertes up to 1.6 cm proximal to the acetabular rim [31].

### Guide wire/screw angles

The angle between the sagittal and coronal plane and the potential screw was a letral angulation of 15–22° and a caudal orientation of 30–35.3°, respectively [49, 50, 55].

For practical purposes, antegrade application is performed using the 2-2-2-20-20 concept for supraacetabular pin fixation [58].

### Corridor width

Screw diameters of 8 mm in men and 6–7 mm in women are possible [57]. According to the tear drop shape of this corridor, the upper widths was 3.63 mm in males and 5.97 mm in females, the middle width was 7.7 mm in males and 9.93 mm in females and at the lower tear drop the widths was 11.93 mm in males and 12.45 mm in females, respectively [29].

Overall, the SAC was found to be smaller in males compared to females (average 15.8 mm vs. 16.2 mm) [14].

### Radiographic views

For the SAC, all possible standard views and their combinations were recommended including the standard PAP, OOV, IOV, COOO, PIV, TLV, COOI, and CIOO [11, 26, 27, 35, 46, 86].

The most relevant views are the COOO, where the corridor imposes as a teardrop figure [23, 24, 57, 64]. The inferior aspect of the teardrop figure is optimally positioned directly tangential to the acetabular roof (sourcil) and the teardrop shape should be as small as possible for optimal identification of the screw entry point. The capsular insertion up to 16 mm proximal to the sourcil should be considered when inserting screws [31]. The classical IOV confirms the optimal placement of the screw superior to the greater sciatic notch.

For intraoperative analysis of the screw pathway several views are recommended (Fig. 17):

- TLV: confirms the screw superior to the greater sciatic notch [57]
- COOO: analysis of axial screw orientation (starting point) within this bony corridor from the PSIS to the AIIS; possible medial/lateral penetration can be excluded
- PIV: screw path lateral to the SI joint
- IOV: screw path superior to the greater sciatic notch and analysis of screw length
- COOI [4]: analysis of the lateral and medial border of the ilium

**Table:** 

The recommended views are the COOO, COOI and IOV [4, 20] and the TLV for implant insertion into the SAC [32]	

The efficiency of this screw corridor was confirmed for LC-2-screws in a biomechanical Tile type C1.1 injury model with an ipsilateral anterior ring fracture reporting that two supraacetabular screws showed comparable stability as two reconstruction plates placed at the iliac crest and the pelvic brim [42]. Also, in a ACPHT fracture model two supraacetabular LC-2 screws were comparable to combined suprapectineal and quadrilateral buttress plating [6].

Scherer et al. described a technique using a transpedicular working cannula [56].

Robotic assistance in the future may reduce the overall ionizing radiation exposure to the patient and the surgical staff [7].

The reverse corridor can be used for S2-alar-iliac screws (S2AI). The S2-alar-iliac (S2AI) screw is an increasingly performed method for spinopelvic fixation in degenerative and trauma situations. The main advantage is its less prominence, and less tissue dissection compared to classical posterior iliac fixation using the retrograde supraacetabular corridor. Landmarks are the posterior superior iliac spine (PSIS), and the sacral laminar slope (a line perpendicular to the sacral laminar slope determines the sagittal screw trajectory) [41].

It traverses three cortices giving strong purchase in both the ilium and sacrum [9, 62].

Typically, the starting point is between the space between the S1 and S2 neural foramina [84].

Description of the insertion point range from a midpoint between S1/S2 foramina and 2 mm medial to the lateral crest to 1 mm inferior and 1 mm lateral to the S1 foramen [33, 89].

Lin et al. defined a mean sagittal screw angle of 44.0° orientated from cranial to caudal and a medio-lateral angle of 37.3° based on CT data [41]. The posterior entry point is located in average 5.9 mm distal to the caudal border of the S1 foramen. The resulting mean screw length was 10 cm. In transitional lumbosacral anatomy, the starting point was 3.4 mm higher.

Other authors reported angulations from 20 to 29° caudally and 30 to 37° laterally based on the individual sacral anatomy [45, 89].

## Modified SAC

Additionally, in some fracture situations, a more anterolateral or even lateral screw insertion is helpful to address oblique fractures or support superior marginal impactions. To confirm these supraacetabular screw placements, for posterior wall fractures, Tosounidis et al. recommended the COOI [69] with an average screw direction of 22° medially directed to the sagittal plane and 35° cranial to the horizontal plane [5, 19].

This so-called “magic screw” starts at the outer iliac table and is directed to the ischial spine for supporting a quadrilateral plate reduction.

## Gluteus medius pillar corridor

**Fig. 20:**
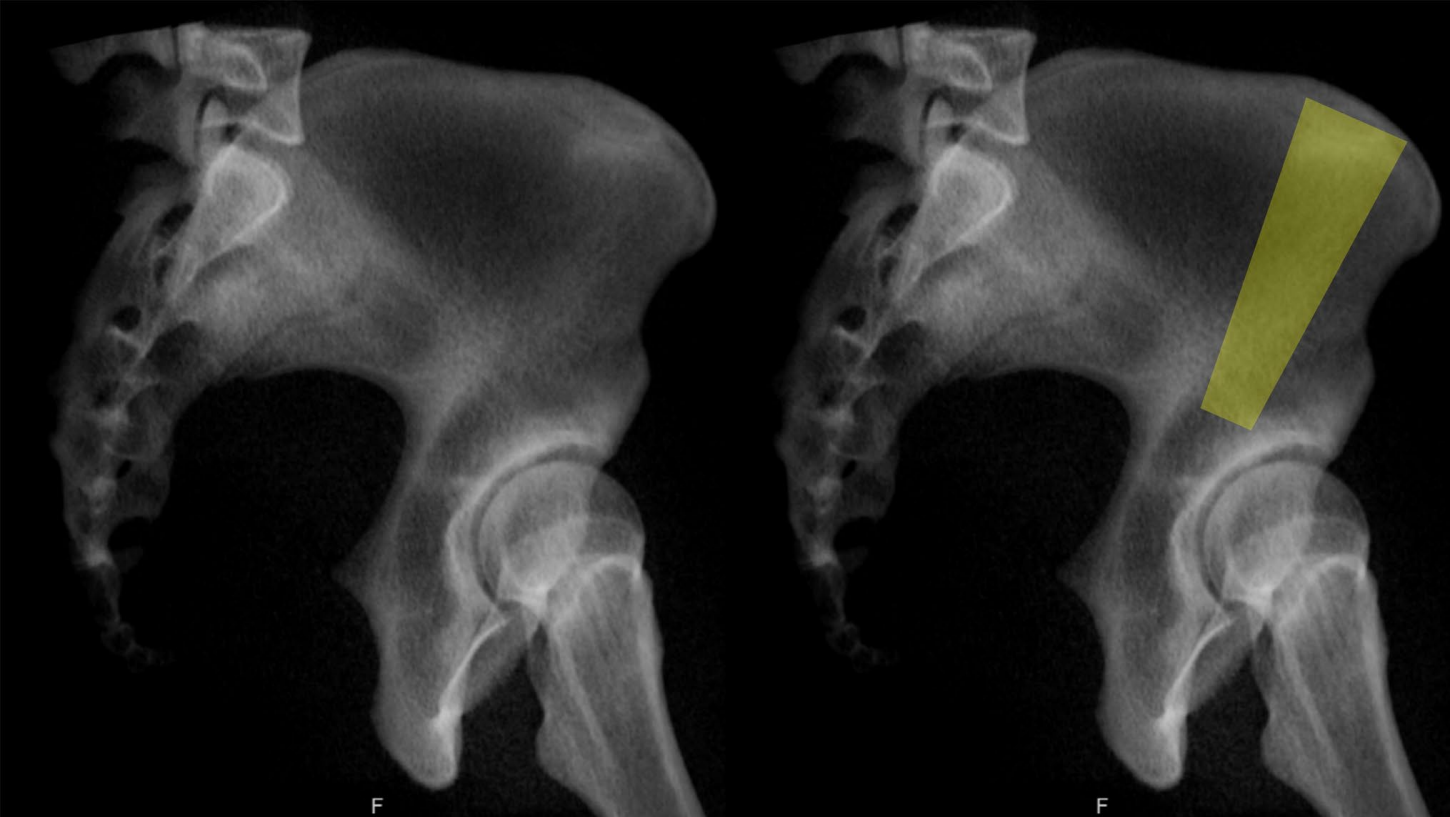
Gluteus Medius Pillar Corridor, starting approx. 4–5cm lateral to the ASIS at the iliac crest and extending to the superior/superoposterior acetabular dome

The thick bone structure with adequate space between both iliac cortices, starting approximately 4–5 cm lateral to the ASIS at the iliac crest and extending to the superior/superoposterior acetabular dome (Fig. 20), is an additional corridor for potential screw placement [4].

In a first analysis, this corridor starts at the iliac crest 5–6 cm posterior to the ASIS and spans 6–8 cm anterior to posterior [54].

This corridor is more often used for Schanz screw insertion for manipulation of anterior column fragments rather than fixation of fractures (Bishop). It is frequently used for the placement of anterior superior pelvic external fixators [61, 71].

The isthmus of this corridor was reported to be 5.3 mm in men and 4.3 mm in women [74]. It was located 27.6 mm in men and 36.3 mm in women caudal to the iliac crest in direction to the ischial tuberosity. No difference with sacral dysmorphism was observed.

## Conclusion

Adequate intraoperative visualization is mandatory for implant application in pelvic ring injuries.

Safe application of screws into the main hemipelvic osseous corridors, e.g. retrograde and antegrade superior ramus/anterior column corridor, supraacetabular corridor and the gluteus medius pillar corridor is possible with detailed understanding of the specific intraoperative radiographic anatomy.

Successful treatment depends on complete preoperative planning and understanding of fluoroscopic imaging based on pelvic anatomy, radiology, and clinical applications of these osseous corridors according to the 3-ring concept of the hemipelvis.

## Data Availability

No datasets were generated or analysed during the current study.
